# A genome-wide comprehensively analyses of long noncoding RNA profiling and metastasis associated lncRNAs in renal cell carcinoma

**DOI:** 10.18632/oncotarget.21206

**Published:** 2017-09-23

**Authors:** Xue Xu, Yongcan Xu, Chuanqin Shi, Baoyu Wang, Xiang Yu, Yanfen Zou, Tao Hu

**Affiliations:** ^1^ Department of Immunology, Binzhou Medical College, Yantai 264003, China; ^2^ Department of General Surgery, Huzhou Central Hospital, Huzhou 313000, China; ^3^ Department of General Surgery, The Affiliated Yantai Yuhuangding Hospital of Qingdao University, Yantai 264000, China; ^4^ Department of Obstetrics and Gynecology, The Affiliated Yantai Yuhuangding Hospital of Qingdao University, Yantai 264000, China

**Keywords:** renal cell carcinoma, lncRNAs profiling, metastasis, prognosis, marker

## Abstract

Recently, a growing number of studies have indicated that long noncoding RNAs (lncRNAs) are emerging as new critical regulators of tumorigenesis and prognostic markers in multiple cancers. However, the expression pattern of lncRNAs and their contributions in renal cell carcinoma (RCC) remains poorly understood. In this study, we performed a genome-wide comprehensively analysis of lncRNAs profiling and clinical relevance to provide valuable lncRNA candidates for the further study in RCC. RCC and non-tumor tissues RNA sequencing data, and microarray data were obtained from The Cancer Genome Atlas (TCGA) and Gene Expression Omnibus (GEO), then, these data were annotated and analyzed to find dysregulated lncRNAs in RCC. We identified that hundreds of lncRNAs were differentially expressed in RCC tissues compared with normal tissues, and genomic variation analyses revealed that copy number amplification or deletion happened in some of these lncRNAs genome loci. Moreover, lots of lncRNAs expression levels are significantly associated RCC patients overall survival time, such as PVT1 and DUXAP8. Finally, we identified some novel metastasis associated lncRNAs in RCC (such as DUXAP8) by analyzing lncRNAs profiling in the RCC tissues from patients with metastasis compared with the primary RCC tissues without metastasis; knockdown of DUXAP8 could impair RCC cells invasive ability *in vitro*. Overall, our findings illuminate a lot of lncRNAs are aberrantly expressed in RCC that may offer useful resource for identification novel prognostic markers in this disease.

## INTRODUCTION

Renal cell carcinoma (RCC) is one of the most common cancers world-wide, and accounts for nearly 90% of all kidney cancers [1, 2]. Over the past two decades, the incidence of RCC has increased and approximately 20% of RCC patients are diagnosed with advanced stage while 30% of RCC patients develop metastasis or local recurrence [3, 4]. Patients with advanced RCC (stage IV) typically respond poorly to chemotherapy and radiotherapy, which resulted in a significantly decreased 5-year survival rate (less than 30%) [5]. Unlike other cancers, there are very few biomarkers for RCC, hence, a better understanding of the RCC pathogenesis and molecular mechanisms underlying RCC metastasis is required to improve early detection and treatment for RCC patients.

In the past years, the achievement and annotation of human whole genome sequence data and ENCODE (Encyclopedia of DNA Elements) data determine that more than 90% of the genome is actively transcribed, but only 2% of the transcripts encodes protein, while the majority of transcripts are referred to as noncoding RNAs including microRNAs and long noncoding RNAs (lncRNAs) [6–9]. Recent studies have revealed that lncRNAs participate in several important biologic processes, including X chromatin imprinting, cell differentiation, nuclear and cytoplasmic trafficking, cell cycle control, cancer cells metastasis and drug resistance [10, 11]. Moreover, increasing evidence indicates that lncRNAs dysregulation play important roles in human diseases, and large-scale RNA sequencing in various cancers revealed that lots of lncRNAs are differently expressed in tumor tissues [12, 13]. Therefore, lncRNAs have emerged as new regulators in tumorigenesis and cancer progression by functioning as oncogenes or tumor suppressors depending on the circumstance. For example, sun and colleagues found that lncRNA HOXA11-AS is over-expressed in gastric cancer and promotes cell proliferation and invasion through scaffolding the PRC2, LSD1, and DNMT1 and functioning as competing endogens RNA for miR-1297 [14].

In case of RCC, several lncRNAs have been reported to be involved in RCC development and progression. For example, MALAT1 was highly expressed in RCC tissues and associated with reduced patient survival, while silencing of its expression inhibited RCC cell proliferation and invasion through interaction with EZH2 and miR-205 [15]. In addition, HOTAIR exerts oncogenic function in RCC cells by regulating HIF-1α/AXL signaling through inhibition of miR-217 [16]; over-expression of lncRNA HEIRCC promotes cell metastasis by inducing epithelialmesenchymal transition [17]. Although few lncRNAs function and underlying mechanisms have been characterized in RCC, the expression pattern and clinical relevance of the majority lncRNAs in RCC remain unknown. To determine the lncRNAs expression and identify RCC associated lncRNAs, we investigated lncRNAs profiling in RCC samples and adjacent non-tumor samples by analyzing TCGA RNA sequencing data and microarray gene profiling datasets from GEO. This study reveals the lncRNAs expression pattern in RCC, which may provide useful candidates for RCC diagnosis and treatment.

## RESULTS

### Identification of altered lncRNAs profiling in RCC tissues

**Figure 1:**
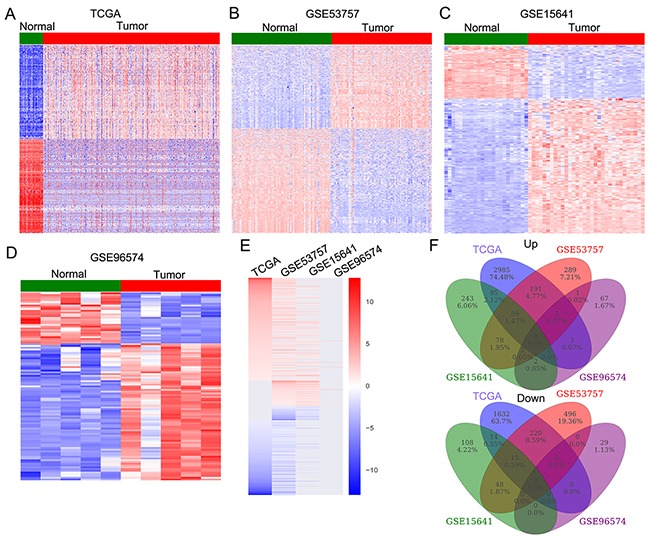
The lncRNAs expression profiling in RCC tissues and non-tumor tissues **(A)** Heatmap of the altered lncRNAs expression in RCC samples and normal tissue samples were analyzed using the TCGA RNA sequencing data. **(B-D)** Heatmap of the dysregulated lncRNAs in RCC were analyzed using the GSE53757, GSE15641, GSE96574 datasets. **(E)** Heatmap of the differentially expressed lncRNAs (consistently altered at least two datasets, fold change) in TCGA, GSE53757, GSE15641, and GSE96574 datasets. **(F)** Venn diagramofaltered of altered profiling in TCGA, GSE53757, GSE15641, and GSE96574 datasets.

To determine the lncRNAs profiling in RCC tissues, we used the TCGA RCC and normal tissue samples RNA sequencing data and 3 microarray gene profiling data (GSE53757, GSE15641, GSE96574) from GEO. The TCGA data consists of 530 RCC samples and 72 normal tissue samples, while the GSE53757 dataset consists of 72 paired samples; GSE15641 consists of 23 Normal, 32 RCC samples; GSE96574 consists of 5 normal samples and 5 RCC samples. Annotation and analyses of these data revealed that 7645 lncRNAs expression were dysregulated in the TCGA dataset (5200 upregulated and 2445 downregulated); 1402 lncRNAs was altered expressed in the GSE53575 dataset (623 upregulated and 779 downregulated); 654 lncRNAs were dysregulated in the GSE15641 dataset (469 upregulated and 185 downregulated); and 107 lncRNAs were differentially expressed in the GSE41657 dataset (78 upregulated and 29 downregulated) (Figure 1A-1D, and Supplementary Table 1). Further overlap analysis showed that 424 lncRNAs were consistently up-regulated and 296 lncRNAs were down-regulated in at least two datasets (Figure 1E-1F, Supplementary Table 2). These data indicates that hundreds of lncRNAs are deferentially expressed in RCC, and part of those altered lncRNAs may be novel biomarkers for RCC diagnosis.

### Genomic alterations of lncRNAs loci in RCC

**Figure 2:**
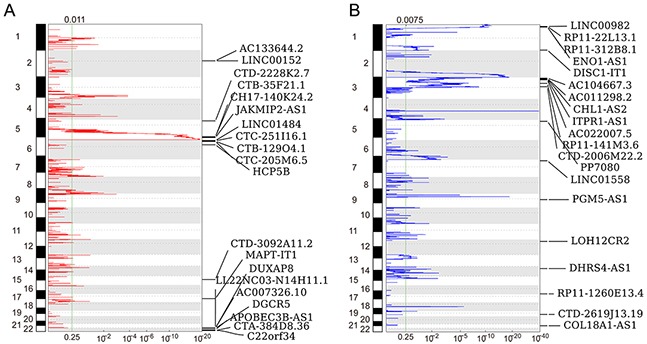
Heatmap of lncRNAs genomic loci copy number variations in RCC **(A)** Frequency of lncRNAs copy number gain (red) in RCC tissues (top20). The each rows represents an lncRNA locus, and are arranged based on the lncRNAs genomic locations. **(B)** Frequency of lncRNAs copy number loss (blue) in RCC tissues (top20). The each rows represents an lncRNA locus, and are arranged based on the lncRNAs genomic locations.

Recent studies have revealed that genomic alterations, transcription factor regulation and epigentic modifications contribute to lncRNAs expression dysregulation in multiple cancer cells. To evaluate whether the genomic alterations involve in lncRNAs dysregulation in RCC, we downloaded the TCGA somatic copy number alterations data. Then, each of those differentially lncRNAs genomic loci SCNAs frequencies were calculated, and alterations occurred in all RCC samples with q value less than 0.25 was defined as significant alteration. The analysis results showed that 63 overexpressed lncRNAs (such as LINC00152, LINC01484 and DUXAP8) with frequency gain and 35 down-regulated lncRNAs (such as LINC00982, LINC01558 and PGM5-AS1) with frequency loss in RCC (Figure 2A and 2B, and Supplementary Table 3). These findings suggest that some of these lncRNAs dysregulation in human RCC tissues is related with genomic somatic copy number variations.

### Identification of RCC survival associated lncRNAs

**Figure 3:**
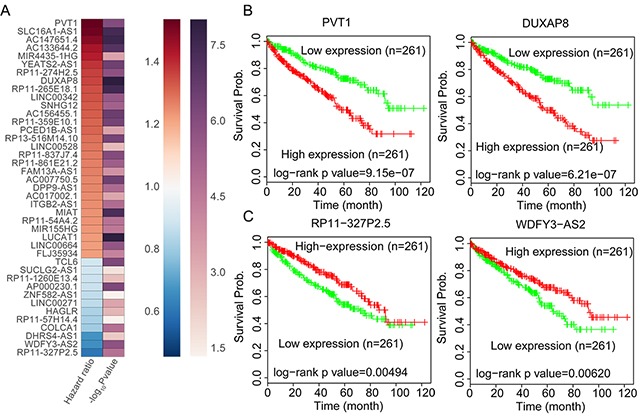
RCC Patients OS associated lncRNAs analysis **(A)** Heatmap of the OS related lncRNAs log rank P value and Hazard ratio value in RCC using the TCGA clinical data. **(B)** The Kaplan-Meier curve for RCC patients OS in higher or lower PVT1 and DUXAP8 groups in the TCGA set was examined using the two-sided log-rank test. **(C)** The Kaplan-Meier curve for RCC patients OS in higher or lower WDFY3-AS2 and RP11-327P2.5 groups in the TCGA set was examined using the two-sided log-rank test.

A growing number of studies have demonstrated that a lot of lncRNAs expression levels are associated with multiple cancers patients prognosis, and those lncRNAs could be used as valuable predictors for patients survival time. To identify RCC patients survival associated lncRNAs, we performed univariable Cox regression analyses using TCGA data. The results of Cox analyses showed that 201 up-regulated lncRNAs and 75 down-regulated lncRNAs are significantly related with RCC patients poorer OS (log rank P<0.05) (Figure 3A Supplementary Table 4). Taken PVT1, DUXAP8, WDFY3-AS2, and RP11-327P2.5 for example, RCC patients with higher PVT1 and DUXAP8 expression levels had shorter OS time, while RCC patients with lower WDFY3-AS2 and RP11-327P2.5 expression levels had shorter OS time (Figure 3B and 3C). These findings indicate that these RCC survival associated lncRNAs may be valuable candidates for RCC patients survival time prediction.

### Identification of metastasis associated lncRNAs in RCC

**Figure 4:**
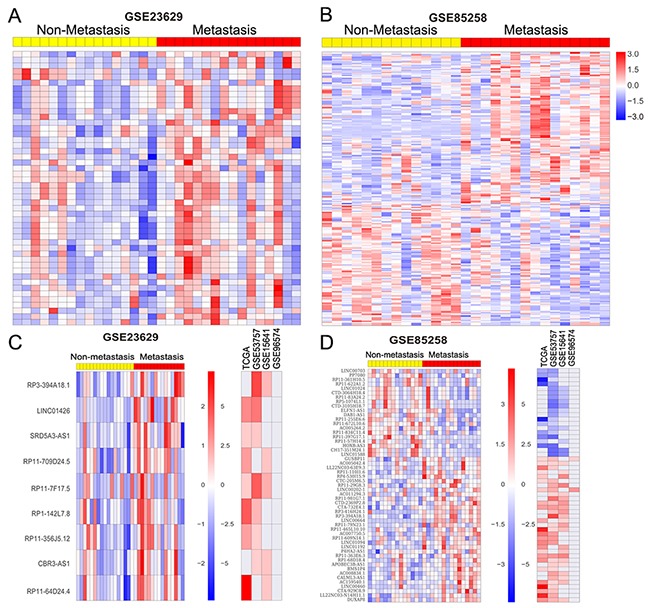
RCC metastasis associated lncRNAs analysis **(A, B)** Heatmap of the altered lncRNAs profiling in metastatic RCC tissue samples compared with primary RCC tissues without metastasis were analyzed using the GSE85258 and GSE23629 datasets. **(C, D)** Heatmap of the significantly upregulated and downregulated lncRNAs in metastatic RCC tissues compared with primary RCC tissues, and their fold-changes in RCC compared normal tissues in TCGA, GSE53757, GSE15641, and GSE96574 datasets.

In RCC, metastasis are detected in approximately 30% of patients, and an additional 30% to 50% of patients with initially localized tumors progress to distant metastases. However, the genetic mechanisms of distant metastasis and RCC progression remains poorly understood. To identify differences in lncRNAs expression associated with RCC increasing metastatic activity, we analyzed lncRNA expression profiling between patient-matched primary and metastatic RCC tumors using two independent data GSE85258 and GSE23629. The GSE85238 dataset contains 15 pairs of primary RCC tumors and patient-matched pulmonary metastases, while GSE23629 contains 16 paired primary RCC tumors and patient-matched metastatic RCC tumor. Analysis of these data revealed that 159 lncRNAs are increased in metastatic RCC tumor while 127 lncRNAs are down-regulated in metastatic RCC tumor compared with primary RCC tumor in GSE85238 dataset (Figure 4A and Supplementary Table 5); 46 lncRNAs are up-regulated in metastatic RCC tumor while 2 lncRNAs are down-regulated in metastatic RCC tumor compared with primary RCC tumor in GSE23629 dataset (Figure 4B and Supplementary Table 5). Among these lncRNAs, 40 lncRNAs are also up-regulated and 20 lncRNAs are down-regulated in at least two datasets from above analysis results (Figure 4C-4D). These findings indicate that lncRNAs dysregulation might also contribute to RCC metastasis and progression.

### Knockdown of DUXAP8 inhibits RCC cells invasion

**Figure 5:**
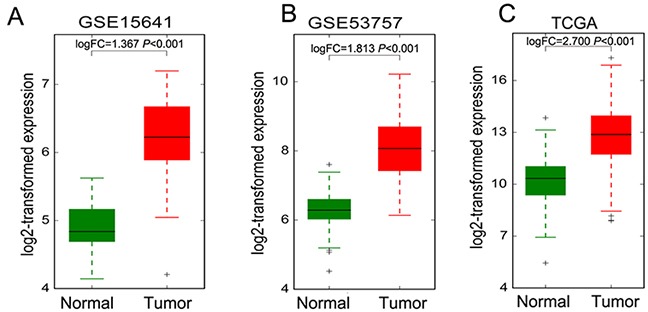
The DUXAP8 expression levels in RCC tissues and non-tumor tissues **(A-C)** The expression levels of DUXAP8 in RCC and normal tissues in TCGA, GSE53757 and GSE15641 datasets.

**Figure 6:**
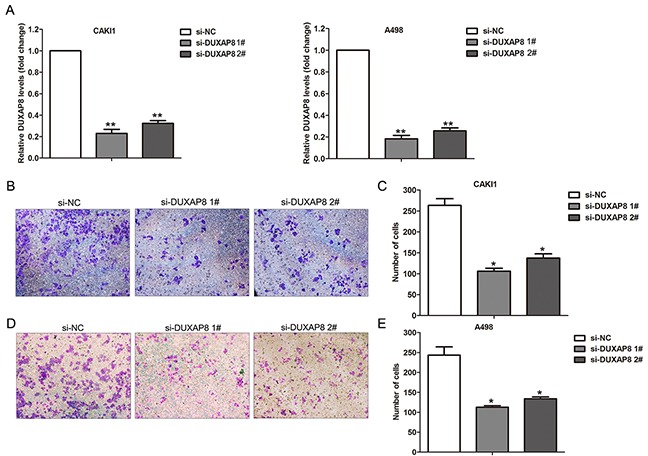
Effect of DUXAP8 knockdown on RCC cells invasion **(A)** The expression levels of DUXAP8 were detected in CAKI1 and A498 cells after transfection with DUXAP8 or negative control siRNAs. **(B, C)** Transwell assays was performed to evaluate the invasive ability of si-DUXAP8 or si-NC transfected CAKI1 cells. **(D, E)** Transwell assays was performed to evaluate the invasive ability of si-DUXAP8 or si-NC transfected A498 cells. ^**^ P<0.01; ^*^ P<0.05.

The above analysis results showed that lncRNA DUXAP8 expression is not only significantly up-regulated in RCC tumors compared with normal tissues, but also increased in metastatic RCC tissues than primary RCC tissues (Figure 5A-5C). Importantly, higher DUXAP8 expression level is related with RCC patients shorter OS time. Therefore, we chose DUXAP8 as candidate to determine whether these metastasis associated lncRNAs affect RCC cells invasive ability. Here, we designed DUXAP8-specific siRNAs and tranfected them into CAKI1 and A498 cells to knockdown its expression. The qRT-PCR results showed that these two siRNAs could significantly decrease DUXAP8 expression level in CAKI1 and A498 cells (Figure 6A). Furthermore, transwell assays showed that knockdown of DUXAP8 could impair CAKI1 and A498 cells invasive ability compared with control cells (Figure 6B-6E). These findings suggest that our analysis data can provide valuable RCC metastasis associated lncRNAs for further study.

## DISCUSSION

RCC pathogenesis and progression involves multistep changes in the gene profiling, and a variety of approaches have been used to uncover the molecular profiles that contribute to RCC development and tumor progression. Moreover, non-coding RNAs profiling as well as gene expression profiling has been found to be useful in the prediction of cancer patients clinical outcome, metastatic recurrence and survival time [18, 19]. During carcinogenesis, genetic alterations could drive tumor evolution toward higher grades of malignancy, however, the extent to how the lncRNAs alterations influence this process remains incompletely understood. In this study, we performed integrated analyses of lncRNAs expression profiles and their genome loci copy number variations, and found that thousands of lncRNAs expression are altered in RCC tissues compared with non-tumor tissues. Moreover, many lncRNAs alteration is associated with genome copy number amplification of loss. In addition, some of these lncRNAs expression levels are significantly related with RCC patients OS time, such as PVT1, DUXAP8, WDFY3-AS2, and RP11-327P2.5. Our findings may provide new insights into the etiology of RCC and valuable lncRNAs candidate list for further investigation of lncRNAs roles in RCC.

Tumor cells metastasis account for the majority of cancer-associated death. In contrast to numerous studies that have revealed the pathogenetic mechanisms of primary tumor formation, the biological underpinnings of tumor cells metastasis remain poorly understood [20]. Recently, increasing evidence has demonstrated that lncRNAs are emerging as critical regulators of cancer cells invasion and metastasis [21]. For example, liu and colleagues found that over-expressed lncRNA HOXA11-AS promotes gastric cancer cells migration, invasion and metastasis *in vivo* through interacting with WDR5 and activating β-actinin transcription [22]. In addition, Sun et al. reported that SPRY4-IT [23] and BANCR [24] could inhibit non small cell lung cancer cells invasion and metastasis *in vivo* by suppressing epithelial-mesenchymal transition process. Moreover, a few RCC metastasis associated lncRNAs and their mechanisms by which they affecting RCC cells invasion and metastasis have been characterized. For example, up-regulated lncRNA RCCRT1 is related with RCC patients lymph node metastasis and distant metastasis and promotes RCC cells migration and invasion [25]; increased HEIRCC promotes RCC metastasis through inducing epithelial-mesenchymal transition [17]. To identify other metastasis associated lncRNAs in RCC, we investigated the lncRNAs expression profiling associated with RCC metastasis by annotating primary RCC tumor samples and paired metastatic RCC samples microarray data. Interestingly, we found some novel lncRNAs that associated with increased metastatic activity in metastatic RCC tumor biopsies, such as DUXAP8.

Recently, Sun and colleagues found that DUXAP8 is significantly up-regulated in human non small cell lung cancer tissues, and knockdown of DUXAP8 expression could inhibits NSCLC cells proliferation, migration, invasion and induces apoptosis *in vitro*. Mechanistically, DUXAP8 interacts with histone demethylase LSD1 and histone methyltransferase EZH2, and thereby represses the tumor suppressors EGR1 and RHOB transcription [26]. Moreover, DUXAP8 was also found to be over-expressed in human gastric cancer, and increased DUXAP8 promoted cells proliferation and invasion through epigenetically silencing PLEKHO1 expression by binding with EZH2 and SUZ12 in gastric cancer cells [27]. These findings as well as our results indicate that DUXAP8 may be an important oncogenic lncRNA in RCC metastasis through interacting with histone modification enzymes and repressing some important cancer cells metastasis regulators expression, and DUXAP8 could be an useful prognostic marker and survival predictor for RCC and other cancers patients. In addition to DUXAP8, there are also some novel lncRNAs that may also involve in RCC tumorogenesis and metastasis, such as LINC00264, LINC00462, and LINC00664, and further investigations are needed to document their function and mechanisms in RCC development and progression.

Taken together, our findings reveal that thousands of lncRNAs were differently expressed in human RCC tissues compared with parental normal tissues. Some of those altered lncRNAs are significantly associated with RCC patients overall survival time, and might play important roles in RCC development, metastasis and progression. In this study, we highlights the lncRNAs profiling in RCC and may provide valuable lncRNA candidates as prognostic markers and potential targets for RCC therapy. The present study also has some limitations, for example, only one lncRNA function was validated in RCC cells, while its underlying mechanism remains unclear, which needs to be further studied by other researchers in the future.

## MATERIALS AND METHODS

### TCGA and public microarray data analysis

The TCGA RCC tissue and normal tissue samples RNA sequencing data and corresponding clinical data were obtained from https://portal.gdc.cancer.gov/. Five public RCC microarray gene profiling datasets (GSE53757 [28], GSE15641 [29], GSE96574, GSE23629 [30] and GSE85258 [31]) were downloaded from the Gene Expression Omnibus (GEO). lncRNAs profiling of GEO microarray datasets was analyzed using the Affymetrix Human Genome U133 Plus 2.0 Array and U133A Array, Agilent-067406 CBC lncRNA + mRNA microarray V4.0. These RNA sequencing and microarray data was preprocessed by using R software and packages.

### lncRNA loci genomic variation analysis

The raw RCC tissues somatic gene copy number variation data was obtained from Broad GDAC FireBrowser website. Next, the significantly recurrent of each lncRNAs genomic regions copy number amplifications or deletions were determined using GISTIC 2.0. All of the lncRNAs genomic loci were mapped the GISTIC peaks. Then, the amplification or deletion peaks with q values <0.25 were considered as significant. The focal/broad frequencies, number of lncRNAs in peaks, and peak q values were summarized at gene level.

### Analysis of survival associated lncRNAs in RCC

To document the relationship between lncRNAs levels and RCC patients overall survival (OS) time, the univariable Cox regression analyses was conducted. Then, the RCC patients were divided into high- and low-expression groups based on the median lncRNAs expression levels. The lncRNAs with log rank *P* value <0.05 between high and low expression groups were defined statistically significant. All of these analysis were conducted using R software and Bio-conductor.

### Cell culture and siRNA transfection

RCC cell line CAKI1 and A498 was purchased from the Type Culture Collection of the Chinese Academy of Sciences (Shanghai, China). CAKI1 and A498 cells was cultured in Dulbecco's modified Eagle's medium (Invitrogen, Carlsbad, CA) with 10% fetal bovine serum (Invitrogen, shanghai, China), 100 U/ml penicillin and streptomycin (Invitrogen), at 37°C with 5% CO2. The DUXAP8 and negative control siRNAs (Invitrogen, Carlsbad, CA) were transfected into CAKI1 and A498 cells using RNAiMAX (Invitrogen) according to the manufacturer's instructions. 48 hours after transfection, the CAKI1 and A498 cells were harvested for RNA extraction. The DUXAP8 siRNA sequences are: siRNA 1#,5′-AAGATAAAGGTGGTTTCCACAAGAA-3′ siRNA 2#, 5′- CAGCATACTTCAAATTCACAGCAAA-3′

### RNA extraction and qRT-PCR

CAKI1 and A498 cells total RNA was extracted using RNeasy Purification Kit (QIAGEN), according to the manufacturer's instructions. Then, 1μg RNA was reverse transcribed into cDNA using PrimeScript RT Reagent Kit (TaKaRa, Dalian, China). SYBR Premix Ex Taq (TaKaRa) was used to detect DUXAP8 expression levels, and GAPDH was used as control. The primer sequence of DUXAP8 is, forward 5′-AGGATGGAGTCTCGCTGTATTGC-3′, reverse 5′- GGAGGTTTGTTTTCTTCTTTTTT-3′. The primer sequence of GAPDH is, forward 5′- AGAAGGCTGG GGCTCATTTG-3′, reverse 5′- AGGGGCCATCCACAG TCTTC-3′. qRT-PCR analysis was performed on ABI7500, and comparative cycle threshold (CT) (2^−ΔΔCT^) method was used to analyze the data.

### Transwell assays

Transwell assays (Corning, Tewksbury, MA, USA) were used to evaluate CAKI1 and A498 cell invasive ability after DUXAP8 or negative control siRNAs transfection. CAKI1 and A498 cells (5×10^4^) in 300 μl medium containing 1% FBS were added into the upper chamber of an insert coated with Matrigel (Sigma-Aldrich). Then, 700μl medium supplied with 10% FBS was placed to the lower chamber. After 24 hours incubation, the CAKI1 and A498 invaded through the membrane were fixed with methanol, stained with 0.1% crystal violet, and then imaged using an IX71 inverted microscope (Olympus, Tokyo, Japan).

### Statistical analysis

The Students t test (2 tailed), and one-way ANOVA were used for qPCR, and *in vitro* assays data analysis using SPSS 17.0 (IBM), R software and Bio-conductor. P value < 0.05 was defined statistically significant.

## SUPPLEMENTARY MATERIALS FIGURES AND TABLES



## Abbreviations

RCC: renal cell carcinoma (RCC)
TCGA: The Cancer Genome Atlas (TCGA)
GEO: Gene Expression Omnibus
lncRNA: long noncoding RNA
SCNA: somatic copy number alterations
OS: overall survival.
