# Supplementary material for: A genome-wide comprehensively analyses of long noncoding RNA profiling and metastasis associated lncRNAs in renal cell carcinoma
Source: Oncotarget. 2017 Sep 23;8(50):87773–81. doi: 10.18632/oncotarget.21206 (PMC5675671; doi:10.18632/oncotarget.21206)
Supplement: Supplementary file 1 [file oncotarget-08-87773-s001.pdf]

A genome-wide comprehensively analyses of long noncoding RNA profiling and metastasis associated lncRNAs in renal cell carcinoma

SUPPLEMENTARY MATERIALS

Supplementary Table 1: The lncRNAs profiling in TCGA and GEO datasets.

See Supplementary File 1

Supplementary Table 2: lncRNAs dysregulation in TCGA and three GEO datasets.

See Supplementary File 2

Supplementary Table 3: The copy number variations of lncRNAs loci in RCC.

See Supplementary File 3

Supplementary Table 4: RCC patients survival associated lncRNAs.

See Supplementary File 4

Supplementary Table 5: RCC metastasis associated lncRNAs.

See Supplementary File 5
